# The growing gap between demand and availability of clinical psychology in Paediatric Gastroenterology: a retrospective analysis of clinical routine care

**DOI:** 10.1007/s00431-020-03825-y

**Published:** 2020-10-16

**Authors:** Eunice Wong, Robert Heuschkel, Caroline Lindsay, Sally Benson, Matthias Zilbauer

**Affiliations:** 1 Department of Paediatrics, University of Cambridge, Hills Road, Cambridge, CB2 0QQ UK; 2 Department of Paediatric Gastroenterology, Hepatology and Nutrition, Cambridge University Hospitals Trust, Level 8 Addenbrooke’s Hospital, Box 116, Hills Road, Cambridge, CB2 0QQ UK; 3 Department of Paediatric Clinical Psychology, Cambridge University Hospitals Trust, Hills Road, Cambridge, CB2 0QQ UK

**Keywords:** Paediatric functional gastrointestinal disease, Functional abdominal pain, Inflammatory bowel disease, Clinical psychology

## Abstract

Clinical psychology intervention in paediatric gastroenterology is vital given the biopsychosocial aetiology of paediatric functional gastrointestinal disorders, and the psychological impact of chronic conditions. The aim was to assess the availability and benefit of clinical psychology in paediatric gastroenterology across the UK and Germany. A retrospective assessment of referrals (*n* = 936 referrals) to clinical psychology was performed at our tertiary paediatric gastroenterology centre between 2010 and 2018. The availability of clinical psychologists and outcome of psychology intervention for children with functional abdominal pain were also assessed. Access to clinical psychology across the UK and Germany was assessed using an online questionnaire. We observed a substantial rise in the number of clinical psychology referrals between 2010 and 2018. Increasing demand was not matched by sufficient increase in availability of clinical psychology, leading to longer waiting times. A major benefit of clinical psychology intervention was highlighted with 95% of patients (*n* = 20) reporting a significant reduction in symptoms. Of the 12 centres who responded, 11 centres have direct access to clinical psychology with a mean of 13% of patients requiring psychology referrals annually.

*Conclusion*: Despite evidence of its benefit and increasing demand, there is insufficient access to clinical psychological services, highlighting the urgent need to address this important issue.

**Table:** 

**What is known:** • *The biopsychosocial pathophysiology of functional gastrointestinal disorders involves a disordered brain-gut interaction, which emphasizes the close link between psychological factors and altered gut function.* • *Psychological intervention, as an adjunct to medical treatment, improves outcomes in paediatric patients with gastrointestinal (GI) disease such as functional gastrointestinal disorders and inflammatory bowel diseases* **What is new:** • *There is a rising number of referrals from paediatric gastroenterology to clinical psychology in our centre which is not met by a sufficient increase in the availability of clinical psychologists. Similarly, access to clinical psychological services is lacking in several paediatric gastroenterology centres in the UK and Germany.* • *Strategic action is required to address this important gap in the care of children suffering from GI diseases.*

**What is known:**

• *The biopsychosocial pathophysiology of functional gastrointestinal disorders involves a disordered brain-gut interaction, which emphasizes the close link between psychological factors and altered gut function.*

• *Psychological intervention, as an adjunct to medical treatment, improves outcomes in paediatric patients with gastrointestinal (GI) disease such as functional gastrointestinal disorders and inflammatory bowel diseases*

**What is new:**

• *There is a rising number of referrals from paediatric gastroenterology to clinical psychology in our centre which is not met by a sufficient increase in the availability of clinical psychologists. Similarly, access to clinical psychological services is lacking in several paediatric gastroenterology centres in the UK and Germany.*

• *Strategic action is required to address this important gap in the care of children suffering from GI diseases.*

## Introduction

Psychological factors have long been recognized for their key role in both causing and/or modulating diseases that affect the gastrointestinal (GI) tract. For example, functional gastrointestinal disorders (FGIDs) as defined by Rome IV are disorders of brain-gut interaction [1], highlighting the direct influence of psychological factors on the onset and exacerbation of FGIDs [3]. FGIDs are now one of the most common diagnoses in paediatric gastroenterology and include functional abdominal pain, chronic vomiting, functional constipation, and functional dyspepsia [2]. Additionally, chronic GI conditions like inflammatory bowel diseases (IBD) have also been shown to be exacerbated by psychological stress [4]. Hence, the role of both psychological assessment and intervention in paediatric gastroenterology has been increasing and integration of clinical psychology into standard care has proven to further reduce symptoms and disability associated with both FGID and IBD [5, 6]. Yet, its importance seems to be insufficiently recognized and there is still a major lack of availability.

The aim of this study was to conduct a service review of our tertiary paediatric gastroenterology centre with regard to the number of referrals to clinical psychology, main GI disorders prompting referral, the benefit of psychological intervention, and the discrepancy between increasing demand and lack of resources in this area. Furthermore, we also assessed current clinical practice in paediatric gastroenterology centres across the UK and Germany to evaluate their access to clinical psychology.

## Methods

### Referrals within Cambridge University Hospitals

Referrals were made from Paediatric Gastroenterology to Clinical Psychology in CUH between January 2010 and December 2018, and information regarding indications and clinical diagnoses were obtained through both an updated spreadsheet, as well as the use of clinical coding on the hospital’s electronic health record system, Epic. These referrals comprised 936 children aged between 1 month and 17 years.

### Availability of clinical psychologists

The availability of paediatric clinical psychology services within the paediatric gastroenterology department at CUH was assessed through calculating the number of ‘psychology hours per week’. This was calculated by calculating the number of psychologists working in our department whilst considering part time employment (i.e. number of days worked per week and/or hours per day) over the years 2013–2018. The number of ‘psychologist hours per week’ was then calculated as the sum of the number of days worked per week by each clinician hired multiplied by 8 (8 h for a full day).

### Assessing the benefit of clinical psychology intervention

A cohort of patients was selected to assess the outcomes of clinical psychology intervention using the following selection criteria: (1) a diagnosis of functional abdominal pain and (2) the referral was made in 2017. Clinical psychology intervention included an initial appointment with the psychologist to undertake a biopsychosocial assessment, followed by follow-up appointments to set and achieve patient-specific goals. Clinical coding on Epic retrieved 30 patients referred with functional abdominal pain in 2017. Among the 30 patients, 10 were excluded as their symptoms improved before their appointment, or they were referred to other services such as Child and Adolescent Mental Health Services. The final cohort of patients comprised 20 children aged 6 to 16 (14 female, 6 male), and the outcome measured was a self-reported improvement in symptoms.

### Questionnaire

An online questionnaire on Google forms was sent out to various centres across the UK and Germany to assess the access to clinical psychology services from paediatric gastroenterology centres (Appendix A).

## Results

### Availability of clinical psychologists insufficient to meet rising referrals

**Fig. 1:**
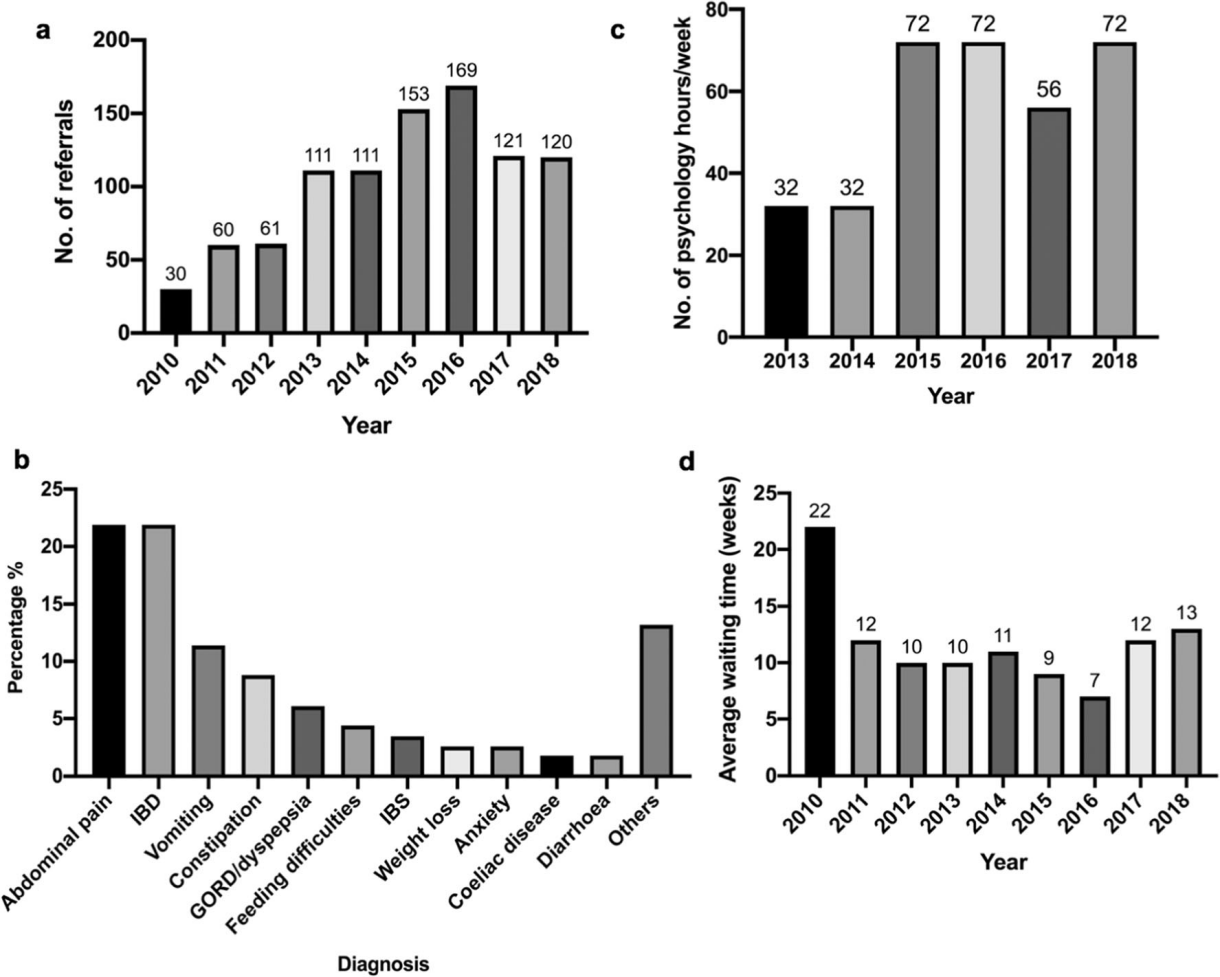
**a** Number of referrals from Paediatric Gastroenterology in CUH to Clinical Psychology from 2010 to 2018. **b** Total number of psychology hours per week worked in the Clinical Psychology department of CUH from 2013 to 2018. **c** Distribution of diagnoses of patients referred from Paediatric Gastroenterology in CUH to Clinical Psychology in 2018 only. **d** Average waiting time to be seen by Clinical Psychology following referral from 2010 to 2018, expressed in weeks

There was a total of 936 referrals made from paediatric gastroenterology to clinical psychology from January 2010 to December 2018, with a mean of 104 referrals made per year (Fig. 1a). The number of referrals per year nearly tripled in 2015–2016 compared with 2010–2012, demonstrating the rising demand for clinical psychology. The 28% fall in referrals from 2016 to 2017 could be attributed to the increase in waiting time in 2017 (Fig. 1d), which may have caused consultants to limit the number of referrals to reduce the pressure on services.

The average waiting time to be seen by clinical psychology decreased from 22 weeks in 2010 to 7 weeks in 2016. This could be explained by a 125% increase in the total number of psychology hours worked per week from 2014 to 2015. However, the average waiting time increased by 71% to 12 weeks in 2017 which is due to a 22% reduction in number of clinical psychology hours per week in 2017.

### Main diagnoses of referrals to clinical psychology

Patients with functional abdominal pain and IBD made up 50% of referrals made to clinical psychology (Fig. 1b). Other functional gastrointestinal disorders which required clinical psychology intervention included functional vomiting, constipation, and gastro-oesophageal reflux disease/dyspepsia. The indications for referral recorded included ‘support’, ‘coping strategies’, and ‘symptom management and understanding’.

### Increasing discrepancy between rising referrals and number of child psychology hours available

The 82% increase in number of referrals made to clinical psychology from 2014 to 2015 (Fig. 1a) was matched by a 125% increase in the total number of psychology hours worked per week from 32 to 72 (Fig. 1c). Although the number of psychology hours worked per week rose back to 72 in 2018, the average waiting time remained high at 13 weeks, probably due to the back log of appointments.

### Assessing the benefit of clinical psychology intervention

The qualitative review of clinical psychology intervention in 20 patients with functional abdominal pain referred in 2017 showed that clinical psychology intervention was very successful, with 19 out of 20 (95%) patients reporting improvement in symptoms. Each patient had an average of 2–3 appointments and was discharged when patient specific goals were met or when symptoms improved.

### Major demand and lack of sufficient access to child psychology in paediatric gastroenterology were recognized in centres across the UK and Germany

The questionnaire was sent out to Paediatric Gastroenterology consultants across the UK and Germany and a total of 12 centres responded. Eight of these centres were tertiary care centres, while 2 were secondary and the remaining 2 were primary care centres. Each centre had a mean of 3 paediatric gastroenterology consultants (SD = 1.62) and a mean of 730 patients (SD = 577.19) seen each year. Eleven of the 12 centres (91%) have direct access to a dedicated paediatric clinical psychology team with an average of 3 (SD = 1.36) clinical psychologists in each team. Of the centres that have direct access to clinical psychology, there was a mean of 95 referrals (SD = 72.58) made to clinical psychology per year, making up about 13% of patients seen per year. The main diagnoses of paediatric gastroenterology patients referred were IBD and FGIDs, while some other diagnoses included eating disorders, autoimmune hepatitis, short bowel syndrome, depression, and anxiety. Half of the centres deemed access to clinical psychology from their department satisfactory, while 2 centres deemed it excellent and the remaining 4 centres inadequate. An average rating of 8.6/10 was given (SD = 0.99) on the importance of clinical psychology in paediatric gastroenterology (Table 1).

**Table 1:** Responses from Paediatric Gastroenterology Centres across UK and Germany on questionnaire evaluating access to and perceived importance of Clinical Psychology intervention in Paediatric Gastroenterology

Question	Response
Direct access to clinical psychology	91% of Paediatric Gastroenterology centres
No. of clinical psychologists	Average = 3
No. of patients seen per year	Average = 730
No. of referrals to clinical psychology per year	Average = 95
Percentage of patients requiring referral to clinical psychology	13%
Main diagnoses of patients referred	IBD and FGID including functional abdominal pain
Evaluation of access to clinical psychology	17% Excellent; 50% Satisfactory; 33% Inadequate
Rating of importance of clinical psychology	Average = 8.6/10

## Discussion

There has been a growing body of evidence proving the role of psychosocial factors in the aetiology, maintenance, and exacerbation of FGIDs [3] as well as the use of psychological services as an adjunct to standard medical treatment in producing more favourable outcomes in FGIDs and IBD [5, 6]. However, despite clear evidence of the imperative role of clinical psychologists in this field, there remains a gap between the need and provision of clinical psychology services in paediatric gastroenterology centres.

In our centre, there has been a rising demand for clinical psychology services over the past decade, of which the commonest indications include FGIDs such as functional abdominal pain and IBD. This rise in demand can be attributed to a rising number of FGID diagnoses in paediatric gastroenterology and an increased clinician awareness of the utility and benefit of clinical psychology services. Furthermore, in addition to FGIDs, most chronic diseases affecting the GI tract such as IBD, irritable bowel syndrome, coeliac disease, or chronic constipation frequently impact on the mental health of patients, thereby negatively influencing disease outcome. While efforts were made to increase the availability of clinical psychologists, waiting times have increased in recent years, proving that paediatric clinical psychology services are overstretched and have been unable to meet the increasing demand.

A qualitative assessment of the benefit of clinical psychology intervention showed that majority of patients treated for functional abdominal pain in 2017 showed improvement of symptoms following an average of 2–3 clinical psychology appointments. This corroborates existing literature on the benefit of integrating clinical psychology services into standard medical care in paediatric gastroenterology [5]. In addition, the experience of our team of clinical psychologists suggests that non-CBT methods of intervention may also be successful.

Our survey of 12 centres in the UK and Germany show that direct access to clinical psychology services is already in place in most centres with an average of 13% of patients with FGIDs and IBD requiring referral to clinical psychology. However, while most centres acknowledge the importance of incorporating clinical psychology services, many centres feel that access to clinical psychology is still lacking. These findings suggest that despite the major differences in healthcare systems between the UK and Germany as well as likely differences between individual centres, the lack of sufficient access to clinical psychology appears to be a common scenario.

There are however several limitations to our study. Failure to receive questionnaire responses from many of the paediatric gastroenterology centres contacted led to a small sample size of only 12 centres. In our centre, the focus was on assessing the outcomes of patients with functional abdominal pain referred in a year, resulting in a small sample size of 20. Future research could extend to a larger sample of patients and assessing the benefit of other methods of psychotherapy on a wider range of conditions. Another limitation of our study is associated with the difficulty in quantifying child psychology in a routine clinical setting. Calculating the number of hours per may can only be considered as an estimation rather than an exact measure. Nevertheless, the comparison over the years combined with raising numbers of referrals clearly illustrates the growing gap between the increasing demand for and relative lack of provision of clinical psychology in our service.

In conclusion, our study shows that while the integration of clinical psychology services into paediatric gastroenterology centres have proven to be important and beneficial, there is still insufficient access to clinical psychology services across paediatric gastroenterology services in the UK and Germany to meet rising demand. Thus, there is an urgent need to address this issue through educating paediatric gastroenterologists, increasing psychologist training capacities, and allocating additional funding to hire more clinical psychology staff, and designing national and local guidance on the appropriate use of clinical psychology services.

## Appendix

### Questionnaire on clinical psychology intervention in paediatric gastroenterology in the UK and Germany

Aim: to assess the presence and extent of clinical psychology support for patients in Paediatric Gastroenterology across the UK and Germany

1. Name and location of service:
2. Is your department a Primary, Secondary, or Tertiary Care provider?
3. How many Paediatric Gastroenterology consultants are there in your department?
4. Give an estimate of the number of patients seen in Paediatric Gastroenterology per year.
5. Does your department have *direct access* to Paediatric Clinical Psychology?

If there IS direct access:

- Is there a dedicated Paediatric Clinical Psychology Team?
- If there is a dedicated team, how many psychologists are there? If there is not a dedicated team, please describe the psychology services you have direct access to.
- Give an estimate of the number of referrals from Paediatric Gastroenterology to Psychology each year.
- What are the main diagnoses of Paediatric Gastroenterology patients who were referred to Psychology?

If there ISN’T direct access:

- Are there any other psychology services available for patients? If so, please give examples.

6. In your opinion, is the access to Clinical Psychology from your Paediatric Gastroenterology department inadequate, satisfactory or excellent?
7. Rate the importance of child psychology support in the management of children with gastrointestinal conditions on a scale of 1 to 10. (1 = not important 10 = extremely important)

## Abbreviations

FGIDs: Functional gastrointestinal disorders
IBD: Inflammatory bowel disease
CBT: Cognitive behavioural therapy
SD: Standard deviation
